# Mapping growth differentiation factor-15 (GDF15)-mediated signaling pathways in cancer: insights into its role across different cancer types

**DOI:** 10.1007/s12672-025-02121-1

**Published:** 2025-03-25

**Authors:** Akhila Balakrishna Rai, Jalaluddin Akbar Kandel Codi, Giridhara Prema Suchitha, Kadabagere Narayanaswamy Hemavathi, Shobha Dagamajalu, Chandran S. Abhinand, Rajesh Raju, Thottethodi Subrahmanya Keshava Prasad

**Affiliations:** 1 Center for Systems Biology and Molecular Medicine [An ICMR Collaborating Centre of Excellence 2024 (ICMR-CCoE 2024)], Yenepoya Research Centre, Yenepoya (Deemed to Be University), Mangalore, 575018 India; 2 Department of Surgical Oncology, Yenepoya Medical College and Hospital, Yenepoya (Deemed to Be University), Mangalore, 575018 India; 3 Center for Integrative Omics Data Science, Yenepoya (Deemed to Be University), University Road, Deralakatte, Mangalore, 575018 India

**Keywords:** GDF15, Cancer, Signaling pathway, Data mining, Bioinformatics, Molecular interactions

## Abstract

Growth differentiation factor-15 (GDF15) is a cytokine/growth factor that belongs to the Transforming growth factor-ß (TGF-ß) protein family. The expression of GDF15 is low in most human organs under normal conditions. GDF15 is a stress-responsive cytokine primarily produced by macrophages in response to inflammatory stimuli. The altered expression of GDF15 is associated with many cancers due to the inflammation caused by the disease. GDF15 triggers the activity through its receptor Glial-derived neurotrophic factor-family receptor α-like (GFRAL) and mediates multiple downstream signaling cascades, which are involved in the progression of cancers. Considering the biological importance of GDF15 in different cancers, we applied data mining techniques to systematically compile and analyze the signaling events associated with GDF15 using NetPath criteria. This resulted in constructing a detailed GDF15-mediated signaling pathway map, enhancing our understanding of its molecular mechanisms in cancer. Furthermore, proteins linked to colorectal and breast cancer identified in our pathway map were cross-referenced with established cancer pathway databases to identify unannotated proteins, highlighting gaps in the current annotations. To investigate potential therapeutic strategies, we performed molecular docking simulations and identified Vitisifuran B as a novel inhibitor that could block the GDF15-GFRAL interaction. These findings suggest that Vitisifuran B could effectively modulate GDF15 signaling, offering a promising avenue for cancer therapeutics. This study underscores the power of computational approaches, such as data mining and molecular docking, in enhancing our understanding of GDF15 signaling in cancer and identifying potential inhibitors for therapeutic development.

**Supplementary Information:**

The online version contains supplementary material available at 10.1007/s12672-025-02121-1.

## Introduction

Growth Differentiation Factor 15 (GDF15), a stress-responsive cytokine, belongs to the Transforming growth factor-β (TGF-β) family of proteins. It is also referred to as macrophage inhibitory cytokine-1 (MIC-1), prostate-derived factor (PDF), NSAID activated 1 gene (Non-Steroidal Anti-Inflammatory Drug-activated gene, NAG-1), placental bone morphogenetic protein (PLAB), and placental transforming growth factor beta (PTGFB) [1, 2]. In humans, the GDF15 is encoded by a gene *GDF15* located on chromosome 19p13.11. The *GDF15* mRNA codes for a precursor protein consisting of 308 amino acids (aa). This includes a 29-aa signal sequence, a 167-aa propeptide, and a 112-aa mature protein [3, 4]. The propeptide of ~ 40 kDa is cleaved by proprotein convertase subtilisin/kexin (PCSK) type 3, 5 and 6 at the N-terminus of the RXXR furine-like cleavage site to form a mature protein (25 kDa). Two mature proteins linked through a disulfide bond to form an active circulating homodimer GDF15 protein [4–6].

Recently, it has been found that GDF15 specifically binds to GDNF family receptor-like (GFRAL), with a co-receptor Rearranged during Transfection (RET) to induce intracellular signaling for the activation [7]. In response to inflammatory stimuli, GDF15 is primarily produced by macrophages [8, 9]. Under normal physiological states, GDF15 mRNA is produced at low levels in various cells and tissues, including the kidney, lung, pancreas, heart, skeletal muscle, adipose tissue, liver, gastrointestinal tract, placenta, and brain. In humans, GDF15 protein expression is notably high in the placenta, moderate in the prostate and urinary bladder, and low in the gastrointestinal tract, pancreas, and kidney, according to data from the Human Protein Atlas [5, 10–12].

GDF15 is found in both the cytoplasm and extracellular matrix (ECM), as well as within the nucleus. It undergoes dynamic movement between these compartments: from the nucleus to the cytoplasm and then into the ECM. The protein chromosomal Maintenance 1 (CRM1) plays a crucial role in exporting GDF15 from the nucleus to the cytoplasm [13]. The human GDF15 promoter contains binding sites for several transcription factors, including p53, early growth response protein 1 (EGR1), cAMP-response element binding protein (CREB), C/EBP homologous protein (CHOP), transcription factor Sp1, and activating transcription factor 3 (ATF3) [10, 14].

Elevated levels of GDF15 are linked to pathological conditions, including tissue damage and inflammation, as well as to the development of cardiovascular diseases, endocrine diseases (diabetes and obesity) and cancer [5, 8, 15–18]. Additionally, this elevated expression is associated with the onset and progression of various cancers such as breast, colorectal, pancreatic, gastric, and prostate [19–24]. Furthermore, serum GDF15 levels as a biomarker are proposed for the early detection and diagnosis of various cancers [15].

Given its diverse range of functions in different tissues and cellular processes, it is critical to understand the process by which GDF15 acts, the receptors with which it interacts, and the resulting signaling events involved. Unfortunately, there is a significant gap in understanding which pathways are activated in specific cell types or conditions. While there are numerous reports describing GDF15-dependent effects in cancer cells, the depth of these effects is complex, diverse, and inconsistent, lacking any clear consensus. Owing to the biological importance of GDF15 in various cancers, we developed a GDF15 signaling pathway map using literature mining to gather the molecular interactions in various cancers. These experimentally reported interactions were systematically integrated by manually annotating research articles from the literature, enabling them to be depicted as a single pathway map. Previously, our group has published pathways such as RANKL/RANK, CCL19/CCL21-CCR7, Elabela, and several other signaling maps [25–27]. Similarly, we generated the GDF15-mediated signaling pathway map to enhance our understanding of its role and contribute to the existing knowledge in cancer contexts.

Functional enrichment analysis is a commonly used approach to examine transcriptomics and proteomics data. However, it is well-known that current annotation systems often exhibit biases, with only a small subset of genes being well-annotated while the majority remain sparsely covered [28]. To illustrate the relevance of our pathway in systems biology, we focused on proteins associated with colorectal cancer (CRC) and breast cancer (BC). The proteins implicated in CRC and BC progression from this pathway map were cross-referenced with widely used cancer pathway databases to confirm their documented roles in the disease and identify gaps in the existing annotations.

We also analyzed metabolomics data of ‘Kanchanara Guggulu,’ a traditional medicine previously reported for its anti-cancer properties [29, 30]. To further investigate its potential in regulating GDF15-induced signaling and anticancer activities, we conducted molecular docking simulations to assess whether its metabolites could effectively target the GDF15-GFRAL binding site and modulate its interaction.

## Methodology

### Pathway map development

We carried out a PubMed search for the research articles pertaining to GDF15-mediated signaling using the search terms ‘(“GDF15” OR “GDF-15”) AND (“signaling” OR “signalling” OR “pathway”) NOT review’ to develop a GDF15 pathway map. The literature was manually screened to select experimental studies that contain information on the downstream signaling events that occur when GDF15 is stimulated. We manually curated the signaling reactions from the studies using previously published PathBuilder, NetPath, and NetSlim annotation criteria [31–33]. The molecular events were grouped into five categories: protein/gene regulation, protein activation/inhibition, enzyme catalysis/post-translational modifications (PTMs), molecular associations, and protein translocation between cell organelles. Other information related to the curation was also included, such as cell lines used in the experiment, experiment type, and PTM sites and residues. Diverse types of molecular reactions reported under the influence of GDF15 thus gathered were manually curated into formatted Excel sheets. Each molecular event in the GDF15 signaling pathway is linked to the respective research article through PubMed identifier. Using known subcellular localizations, signaling contexts and molecular interactions of these proteins, a signaling pathway map was manually drawn using the PathVisio tool [34] that provides the gpml format of the pathway reactions. Different types of edges were employed to represent various reaction types, with dashed lines indicating phosphorylation and inhibition, while solid lines represent gene and protein regulation. Post-translational modifications (PTMs), along with their respective residues and interacting molecules, are highlighted using specific colors. A detailed legend was included to facilitate a clearer understanding of the pathway map. The pathway reactions and the pathway map were reviewed by internal reviewers (S.D., R.R., and T.S.K.P.), with expertise in developing pathway maps for numerous ligand-receptor systems [25, 26, 35–39].

### Annotation of GDF15 as a biomarker in the pathway map

Following the construction of the GDF15 pathway map based on experimental data, we conducted a biocuration of published proteomic datasets to assess the role of GDF15 in cancer. In cases, where GDF15 was altered in cancers, we marked it with a star symbol (*) on the pathway map to visually highlight its relevance to cancer biology.

### Comparison with pathway databases and TCGA mutation data

In addition, we compiled the proteins associated with the CRC pathway, including GDF15, VIM, CDH1, CDH2, AKT1, TGFBR2, SMAD2, MAPK1, MAPK3, MAPK14, among others. These proteins were analyzed using STRING database (https://string-db.org/) (Szklarczyk et al., 2023) to visualize their molecular interactions and their roles in CRC, leveraging widely available pathway databases like KEGG and WikiPathways. Additionally, we compared these findings by analyzing the colon adenocarcinoma TCGA data using SRplot’s MAF Oncoplot tool (https://www.bioinformatics.com.cn/plot_basic_maf_oncoplot_135_en). A similar analysis was performed for the proteins associated with the breast cancer pathway to provide deeper insights.

### Identification of signature metabolites in Kanchanara Guggulu using a metabolomics approach

We analyzed the raw metabolomics data from ‘Kanchanara Guggulu,’ a formulation previously reported for its anticancer properties, to identify signature metabolites targeting GDF15. The data analysis process is detailed as follows. Mass spectrometry data, both in positive and negative ion modes, was processed separately using MZmine (Version 2.53) [40]. Initially, mzML files were generated from the.wiff files using MSConvert and uploaded into MZmine to extract retention time (RT), *m*/z values, and peak areas of the detected features. Mass detection was performed at MS1 and MS2 levels with predefined intensity thresholds. Precursor ions, along with their fragment details, were selected using the MS/MS peak list builder to generate the *m*/*z* feature list. Features within a 0.05 Da *m*/*z* tolerance were processed through chromatogram deconvolution using the Peak Extender algorithm. During deconvolution, the noise peak height was set to 1.5 × 10^2^, and retention time and m/z tolerances for MS2 pairing were configured to 1 min and 0.1 Da, respectively.

The deconvoluted features were then processed using the isotopic peaks grouper algorithm, with m/z and retention time tolerances set at 0.25 Da and 0.2 min, respectively. These features were subsequently aligned using the Join Aligner algorithm. The features were then gap-filled using the Peak Finder algorithm, with an intensity tolerance of 30%, a retention time tolerance of 0.6 min, and an *m*/*z* tolerance of 0.05 Da. Duplicate peaks were removed using the New Average mode in the Duplicate Peaks Filter algorithm, applying *m*/*z* and retention time tolerances of 0.1 Da and 0.2 min, respectively. The results, including peak areas, RT, m/z values, and feature IDs, were exported as CSV files at the MS2 level. Additionally, precursor masses and their associated fragment details were exported for metabolite assignment. The files obtained after MZmine analysis were further analyzed to obtain MS2-level metabolites using the MS2Compound tool [41]. Metabolites were annotated and downloaded from PlantCyc and HMDB (Human Metabolome Database) [42, 43]. The identified MS2 metabolites were then further evaluated using MS2query [44], which compares library and query spectra through a random forest model. A prediction score greater than 0.7 was set as the cut-off for identifying the best-matching metabolites. The top-scoring metabolites from MS2query, along with rank one metabolites from MS2Compound, were selected for docking.

### Molecular docking screens

**Fig. 1:**
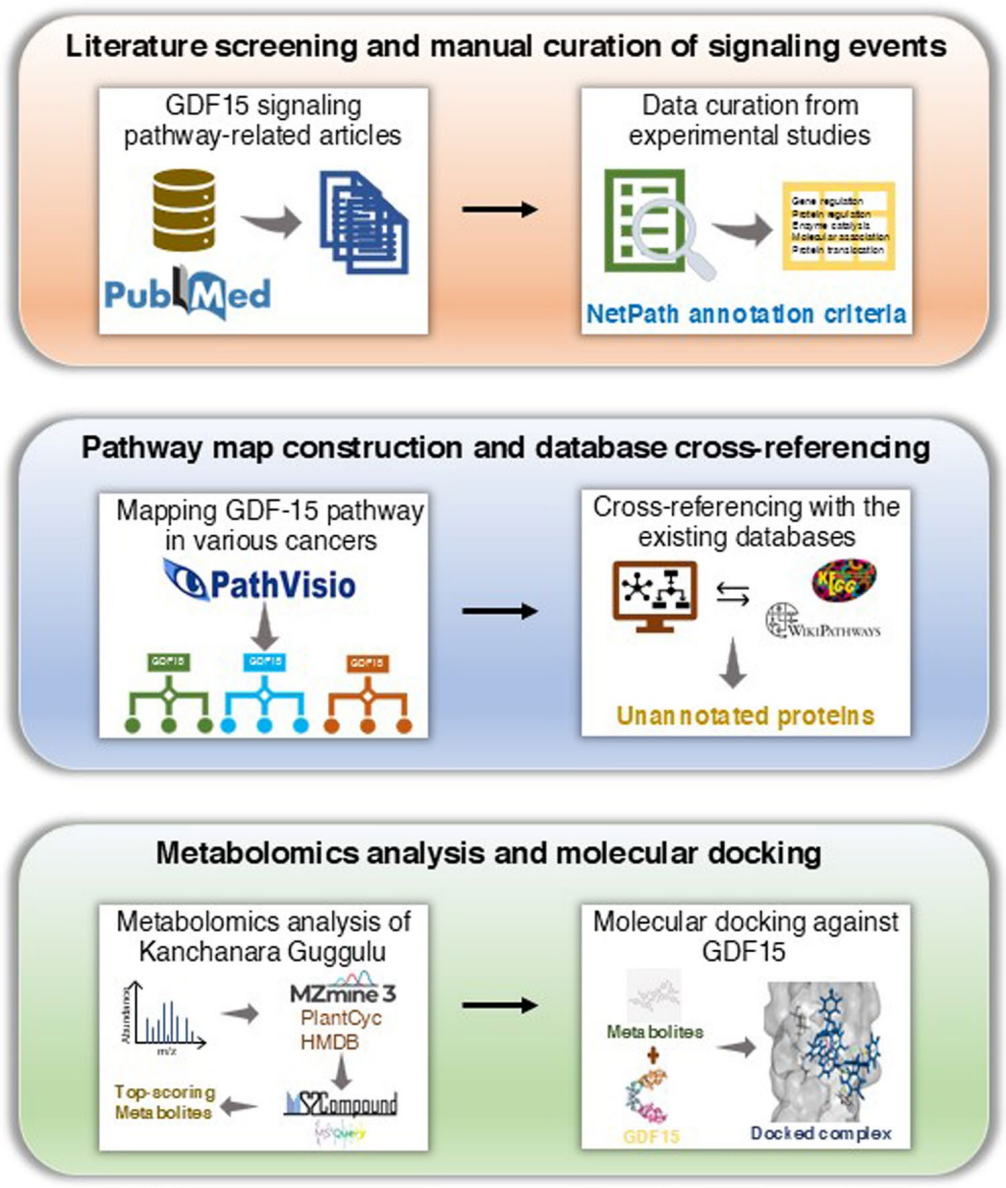
Schematic representation of the workflow adapted in this study. An overall workflow illustrating the key steps: 1. Literature screening and manual curation of signaling events, 2. Pathway map construction and cross-referencing with databases, 3. Metabolomics analysis and molecular docking

The top metabolites of ‘Kanchanara Guggulu’ identified in the analysis were docked against GDF15 to assess whether these metabolites bind to the target protein. The 3D structure of GDF15 (PDB ID: 5VZ4) was obtained from the Protein Data Bank and prepared by removing water molecules and other heteroatoms using the “Prepare Protein” protocol in Discovery Studio 2022. The structures of the metabolites were downloaded from the PubChem compound database and underwent ligand preparation using the ligand preparation protocol in Discovery Studio 2022 before performing molecular docking. The key residues in GDF15 that showed interactions with its receptor were selected and defined as the active site of the protein. Protein–ligand docking was performed using the LibDock protocol in Discovery Studio 2020, with the LibDock score and intermolecular interactions between the ligands and GDF15 considered for selecting the best ligands from the library. Further, some standard chemotherapeutic drugs such as Doxorubicin (PubChem ID: 31703), Paclitaxel (PubChem ID: 36314), Cisplatin (PubChem ID: 441203), and 5-fluorouracil (PubChem ID: 3385) were also checked for their possible interactions with GDF15. The Online Resource 3 table provides the list of software, tools and databases used in each step of the study. The overall workflow of the study is depicted in Fig. 1.

## Results and discussion

**Fig. 2:**
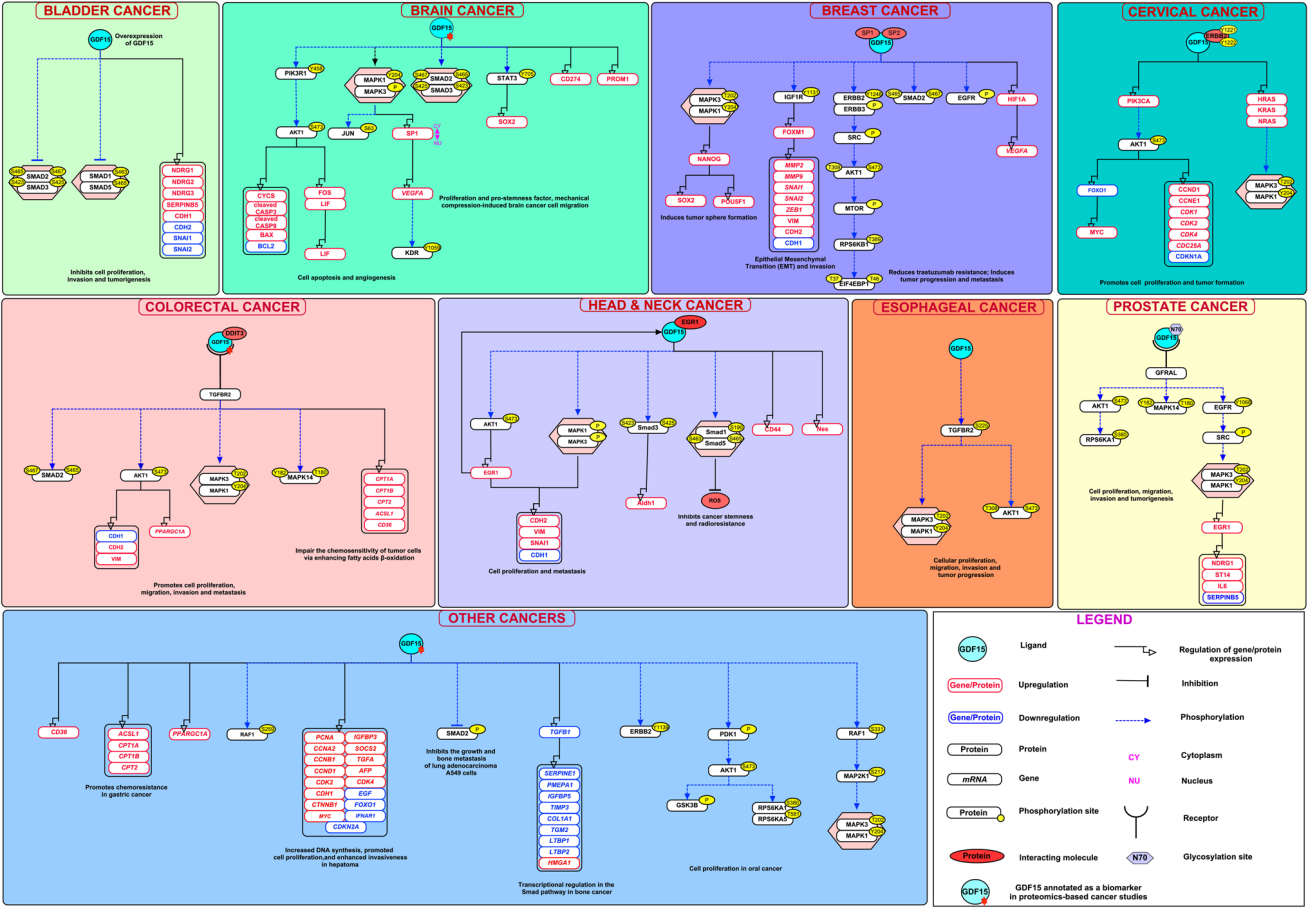
Illustrative depiction of the GDF15-induced signaling pathway in cancer. The signaling pathway diagram depicts the molecules involved in interactions between ligands and receptors, as well as downstream molecular processes triggered by GDF15. These processes encompass various events like molecular association, enzyme catalysis, translocation, and gene regulation. Additionally, details concerning posttranslational modification sites, residues and reporting as biomarker are provided within the pathway

The PubMed search with the specified search terms fetched 645 articles related to GDF15 signaling. Manual screening was performed to select the research articles with specific information about GDF15 signaling in cancer. The manual curation of these chosen articles had 149 molecules grouped into nine activation/inhibition events, six molecular associations, 34 enzyme catalysis, and two translocation events. A total of 52 genes and 46 proteins had differential mRNA and protein expression, respectively (Online Resource 1). These events were merged into a single pathway map. To the best of our knowledge, this resource offers a compilation of diverse molecular reactions facilitated by GDF15 in different cancer conditions, illustrated in Fig. 2.

### A concise overview of the GDF15 signaling map in different cancers

GDF15 is involved in regulating body weight and food intake under physiological and pathophysiological conditions. In normal conditions, the expression of GDF15 remains low, whereas it increases in diseased conditions. GDF15 binds to GDNF family receptor-like (GFRAL) and Rearranged during Transfection (RET), which triggers various downstream signaling cascades. Its impact has been extensively documented across various human cancer types [7, 11, 45]. Stimulation by GDF15 alters the expression of multiple molecules within cells, consequently promoting cell proliferation in prostate cancer, oral cancer, cervical cancer, hepatoma, and bladder cancer cells [46–50]. The significant role of GDF15 signaling in different cancers is described below.

### Head and neck cancer (HNC)

The elevated expression of GDF15 in head and neck cancer tumor tissues and cell lines is involved in tumor stage, lymphovascular invasion, and tumor grade [51]. Li et al. reported that GDF15 contributes to radioresistance and promotes cancer stemness through the inhibition of ROS, upregulation of SMAD1, SMAD5, SMAD3, NES, ALDH1 and CD44 via phosphorylation of SMAD1 (Ser190), SMAD5 (Ser463/465), SMAD3 (Ser423/425) in xenografted tumors in BALB/c nude mice. This demonstrated that overexpression of GDF15 induces significant resistance to radiation treatment [52]. Jin et al., reported that the ectopic expression of EGR1 significantly increased the expression of GDF15, which in turn decreased the expression of CDH1 and increased the expression of CDH2, VIM, and SNAI1 through the activation of AKT1 (Ser473) and MAPK3/1 pathways, which contributes to cancer progression in rhGDF15 treated HNC KB and FADU cells. This study suggested that the GDF15-EGR1 signaling axis may be a potential therapeutic target in HNC patients [51].

### Brain cancer

GDF15 is involved in enhancing the proliferation, invasion and secondary tumor forming behavior through the phosphorylation of MAPK3/1 (Thr202 /Tyr 204), and JUN pathways in brain cancer cells such as H4 and A172[53]. In glioblastoma multiforme (GBM) cells, GDF15 induces phosphorylation of SMAD2 (Ser465, Ser467) and SMAD3 (Ser423, Ser425), thereby upregulating CD274, which regulates tissue development and cancer [54]. Zhu et al., reported that GDF15 induces the phosphorylation of STAT3 (Tyr705), MAPK3, and MAPK1 and the molecular association between FOS and LIF promoter. This leads to the upregulation of LIF, FOS, PROM1, and SOX2 in GSC (glioma stem cells)-like cells (GSCLCs) and U87 TS cells, which promotes glioma stem cell-like phenotype [55]. rhGDF15 induces the MAPK1 (Tyr204) phosphorylation, elevated expression of SP1 and its translocation to the nucleus, where it binds to the promoter region of VEGFA. This results in the upregulation of *VEGFA*, which in turn leads to the phosphorylation of KDR (Tyr1059) and induces angiogenesis in glioblastoma on endothelial cells [56]. Another study reported that GDF15 in glioblastoma cells induces the upregulation of cleaved CASP3, cleaved CASP9, BAX, CYCS and downregulation of BCL2 through the phosphorylation of PIK3R1 (Tyr458), AKT1 (Ser473), SMAD2 (Ser465, Ser467), and SMAD3 (Ser423, Ser425) pathways, which promotes apoptosis in glioblastoma cells [56].

### Bladder cancer

The overexpression of GDF15 in bladder cancer cells leads to the increased level of NDRG1, NDRG2, NDRG3, SERPINB5, and CDH1 and attenuated CDH2, SNAI2, SNAI1 expression, which inhibit the cell proliferation, invasion, and tumorigenesis [50]. The study by Hou et al. reported that the rhGDF15 treatment in bladder carcinoma cells promotes cell proliferation and invasion by the upregulation of NDRG1 and SERPINB5 proteins [57].

### Breast cancer

In breast cancer cells, GDF15 overexpression induces phosphorylation of IGF1R (Tyr1131) resulting in the downregulation of CDH1 and upregulation of CDH2, VIM, FOXM1, IGF1R, *SNAI1*, *ZEB1*, *SNAI2*, *MMP2*, *MMP9*, which mediates epithelial-mesenchymal transition (EMT) and invasion [58]. HER2-overexpressing breast cancer cells (BT474 cells) were stimulated with recombinant human GDF15, which induces phosphorylation of ERBB2 (Tyr1248), AKT1 (Ser473), MAPK3/1 (Thr202 /Tyr204), SMAD2, and SRC leads to trastuzumab resistance [59]. Sasahara et al. (2017) reported that GDF15 induces the phosphorylation of SMAD2 (Ser465/467), MAPK3/1 (Thr202 /Tyr 204). This phosphorylation upregulates POU5F1, SOX2, and NANOG in MCF7 (breast cancer) cells, which maintains cancer stem-like phenotype in breast cancer [60]. The overexpression of GDF15 in MCF7 (breast cancer) cells causes the activation of the MAPK3 pathway [61]. GDF15 in breast cancer cells induces the phosphorylation of AKT1 (Ser473 and Thr308), MAPK3/1 (Thr202 /Tyr204), Tyr phosphorylation of EGFR, ERBB2, ERBB3, MTOR (Ser2448), RPS6KB1 (Thr389), EIF4EBP1 (Thr37/46), and increased expression of *VEGFA*, HIF1A, which are involved in tumor progression and metastasis [62].

### Cervical cancer

In cervical cancer HeLa and SiHa cells, GDF15 interacts with ERBB2 and phosphorylates AKT1 (Ser473), MAPK3/1 (Thr202 /Tyr204), ERBB2 (Tyr1221, Tyr1222). It further induces upregulation of *CDK1*, *CDC25A*, *CDK2*, *CDK4*, CCND1, CCNE1, MYC, PIK3CA, HRAS, KRAS, NRAS and downregulation of *CDKN1A*, CDKN1A, FOXO1, which promotes proliferation of cells [48].

### Colorectal cancer

In colon cancer cells, GDF15 induces phosphorylation of MAPK14 (Thr180, Tyr182), MAPK3/1 (Thr202 /Tyr204), and AKT1 (Ser473), which promotes cell proliferation, migration, and invasion in colon adenoma and colorectal cancer cell lines. The increased GDF15 expression is observed in primary normal colon tissue from people at increased risk for CRC [63]. Zheng et al. (2019) reported that DDIT3 binds to the promoter of GDF15 in hypoxia-induced colorectal cancer cells. It induces downregulation of CDH1 and upregulation of CDH2, VIM and *PPARGC1A*, *CPT1A*, *CPT1B*, *CPT2*, *ACSL1*, *CD36*, genes which are involved in metastasis [64]. Another study reported that GDF15 induction in DLD1 (human colon cancer) cells leads to the upregulation of *ACSL1, CPT1A*, *CPT1B*, *CPT2*, *CD36*, and *PPARGC1A*, which stimulates tumor chemoresistance [65]. Lee et al. showed that GDF15 induces the phosphorylation of SMAD2 (Ser465/467) in recombinant GDF15 added TGFBR2 expressing HCT116 cells [66]. The recombinant rhGDF in HCT116 p53 wild-type cells induces phosphorylation of AKT1 (Ser473), which is involved in the inhibition of drug-induced cell death [67].

### Esophageal cancer

In esophageal cancer cells, the high expression of GDF15 induces phosphorylation of AKT1 (Ser473, Thr308), and MAPK3/1 (Thr202 /Tyr204), which is involved in tumor progression [68]. Okamoto et al. (2020) reported that GDF15 stimulation in TE-8 and TE-11 (esophageal squamous cell carcinomas (ESCC)) cells induces the phosphorylation of AKT1 (Ser473), MAPK3/1 (Thr202 /Tyr204), TGFBR2 (Ser225) and promotes progression of cancer [69].

### Prostate cancer

In prostate cancer cells, overexpression of GDF15 induces the upregulation of ST14, NDRG1, and IL6; and the downregulation of SERPINB5, which are involved in cell proliferation, invasion, and tumorigenesis [46]. Wang et al., reported that prostate cancer cells disseminate to bone, where they interact with osteocytes and release GDF15 into the bone microenvironment. GDF15 binds to its receptor GFRAL on prostate cancer cells, triggering the upregulation of EGR1, which contributes to cancer progression in the bone microenvironment and promotes bone metastasis [70]. In human prostate adenocarcinoma tissue, GDF15 induces the phosphorylation of SMAD2 (Ser433), SMAD3 (Ser435), MAPK3/1 (Thr202 /Tyr204), RPS6KA1 (Ser380), MAPK14 (Tyr182, Thr180), which are involved in cell growth and tumor progression [24, 71]. In agreement with this, Wang et al., reported that the overexpression of the wild-type GDF15 increases the phosphorylation of EGFR (Tyr1068), SRC, MAPK3/1 (Thr202 /Tyr204), and AKT1 (Ser473) level along with cell survival in castration-resistant prostate cancer (CRPC) cells, whereas the N70 glycosylation of GDF15 abolishes the inhibitory effect of GDF15 on EGFR [72].

### Other types of cancers including gastric, hepatoma, lung, bone and oral

In gastric cancer cells, GDF15 induces the FAO (Fatty acid oxidation) associated upregulation of *CD36*, *ACSL1*, *PPARGC1A*, *CPT1A*, *CPT1B*, and *CPT2*, which promotes chemoresistance [73]. The higher expression of GDF15 in HCV-infected hepatoma cells (Huh7.5.1 cells) causes the phosphorylation of AKT1 (Ser473), RAF1 (Ser259), and GSK3B (Ser9). It results in the upregulation of *PCNA*, *CCNA2*, *IGFBP3*, *CCNB1*, *CDK2*, *CDH1*, *CTNNB1*, *MYC*, *CCND1*, *SOCS2*, *TGFA*, *AFP*, *CDK4* and downregulation of *EGF*, *FOXO1*, *IFNAR1*, *CDKN2A*, which are involved in increased DNA synthesis, promoted cell proliferation, and importantly enhanced invasiveness of the cells [49]. The expression of GDF15 in A549 (lung adenocarcinoma) cells inhibits phosphorylation of SMAD2 which in turn is involved in the inhibition of bone metastasis [74]. In human bone osteosarcoma epithelial cells (U20S), GDF15 controls the transcriptional regulation of Smad pathway by the upregulation of *HMGA1* and downregulation of *COL1A1*, *TIMP3*, *TGM2*, *TGFB1*, *LTBP1*, *LTBP2*, *SERPINE1*, *PMEPA1*, *IGFBP5* in human bone osteosarcoma epithelial cells (U20S) [13]. Zhao et al., reported that GDF15 overexpression in oral squamous cell carcinoma (OSCC) cells and xenograft mice model increases the phosphorylation of ERBB2 (Tyr1139), AKT1 (Ser473), MAPK1 (Tyr204), MAPK3 (Thr204), PDK1, GSK3B, RAF1 (Ser331), MAP2K1, RPS6KA1, RPS6KA5, which promotes cellular proliferation [47].

### Common downstream pathways of GDF15 in various cancers

**Fig. 3:**
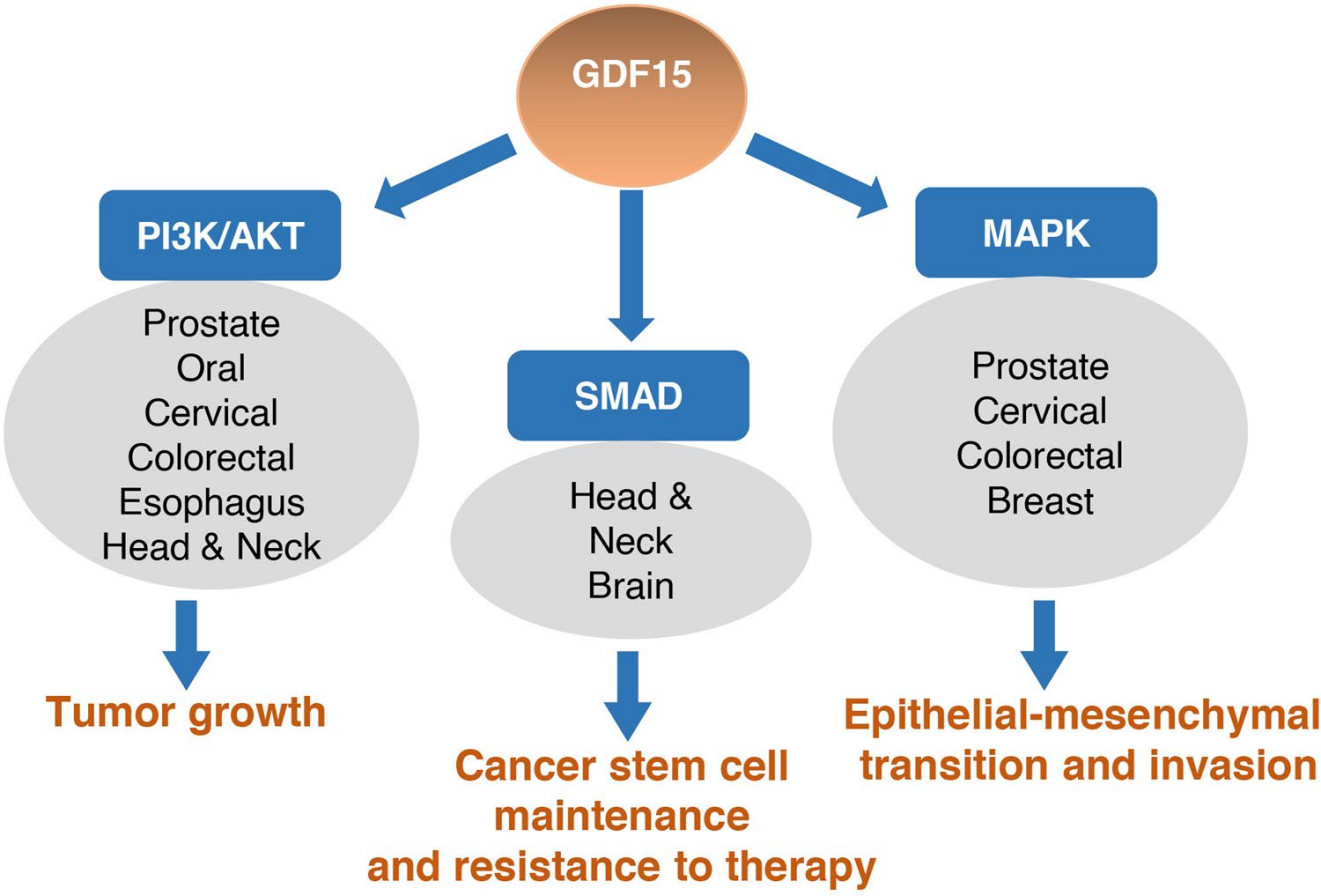
Common downstream pathways of GDF15 in cancer. The schematic representation illustrates the key molecular pathways activated by GDF15 and their role across various cancers

Across different cancers, GDF15 predominantly activates pathways that promote cell proliferation, survival, and metastasis. One of the central themes is the activation of the PI3K/AKT pathway, which is frequently upregulated in cancers including head and neck, prostate, oral, cervical, esophagus, and colorectal. For instance, GDF15 stimulation leads to the phosphorylation of AKT1 (Ser473) and MAPK3/1 (Thr202/Tyr204) in prostate, cervical, and colorectal cancers, facilitating tumor growth and resistance to treatment. Similarly, in breast cancer, GDF15-induced phosphorylation of IGF1R (Tyr1131) and MAPK3/1 (Thr202/Tyr204) supports EMT and invasion. GDF15 also affects the SMAD signaling pathways, which are crucial for cellular responses to TGF-beta superfamily members. In various cancers, such as head and neck cancer and glioblastoma, GDF15-induced phosphorylation of SMAD2 and SMAD3 contributes to cancer stem cell maintenance and resistance to radiation therapy **(**Fig. 3**)**.

Overall, the diverse roles of GDF15 across different cancers reflect its potential as both a biomarker and a therapeutic target. The activation of common pathways such as PI3K/AKT and SMAD, coupled with cancer-specific effects, underscores the complexity of GDF15 signaling. Understanding these mechanisms can provide insights into developing targeted therapies and improving treatment outcomes for cancer patients.

### Emphasizing the significance of the GDF15 pathway

**Fig. 4:**
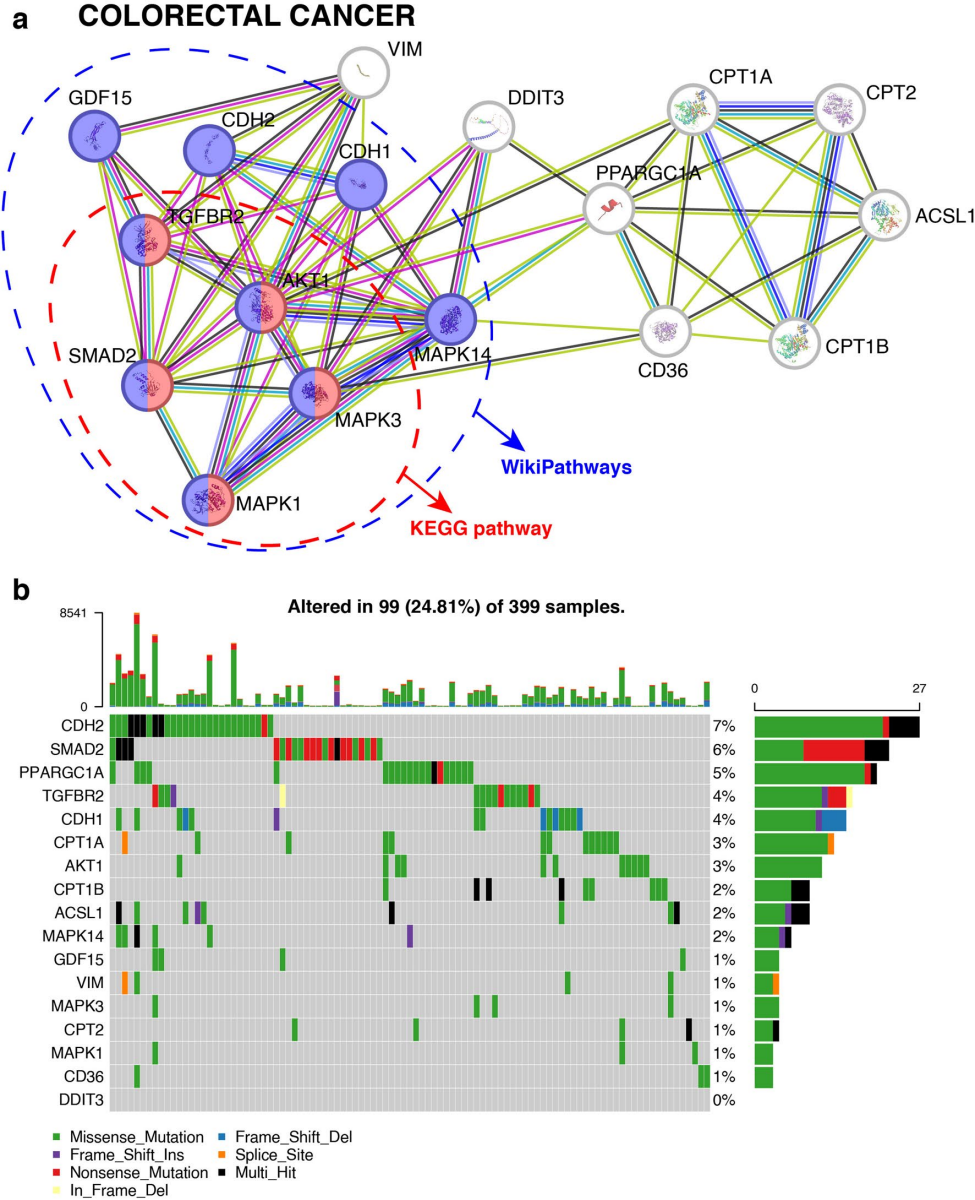
Comparison of GDF15 pathway proteins associated to colorectal cancer with existing pathway databases and TCGA mutational data. **a** The protein interaction network of proteins from the GDF15 pathway map with those found in the colorectal cancer (CRC) pathway. Proteins marked with circles represent hits in KEGG (red) and WikiPathways (blue). **b** The MAF Oncoplot illustrates mutation frequencies of CRC proteins in TCGA data for colon adenocarcinoma

To highlight the significance of the GDF15 signaling pathway, we selected the proteins involved in the CRC and BC pathways. While some key proteins like TGFBR2, AKT1, SMAD2, MAPK3, and MAPK1 were identified in the KEGG pathway database for CRC, additional proteins related to EMT in CRC were found in WikiPathways **(**Fig. 4a**)**. Despite this, proteins such as PPARGC1A, CPT1A, CPT1B, ACSL1, VIM, CPT2, CD36, and DDIT3 did not show hits in any pathway databases for CRC. Similarly, for BC, proteins such as MTOR, ERBB2, MAPK1, RPS6KB1, MAPK3, EGFR, IGF1R, and AKT1 were identified in both KEGG and WikiPathways (Figure S1a). In contrast, proteins like MMP9, CDH1, CDH2, and SMAD2 did not align with any pathway database. This comparison revealed that many proteins curated in this GDF15 pathway map are absent in existing databases, suggesting a gap in existing pathway annotations.

Moreover, we examined the presence of mutations in these proteins related to colon adenocarcinoma and breast invasive carcinoma in TCGA data. Notably, proteins like PPARGC1A and CPT1A were found to be mutated in CRC according to TCGA data, as depicted through an MAF oncoplot (Fig. 4b). Based on TCGA data, proteins such as CDH1 and ERBB3 were found to harbor mutations in BC (Figure S1b). This indicates that while these proteins are absent from pathway databases, they are represented in mutation datasets, further validating their importance in CRC and BC, as reflected in our pathway map. These pathway databases lead to the under-representation of clinically significant genes. The inclusion of poorly annotated but relevant proteins in our pathway map not only enhances our understanding of the targets but also underscores the need for more comprehensive pathway databases.

### Molecular docking of GDF15 with metabolites of Kanchanara Guggulu

Mass spectrometry-based untargeted metabolomics of ‘Kanchanara Guggulu’ enabled the identification of 497 nonredundant metabolites at the MS1 level corresponding to 253 in positive and 244 in negative modes. There were 95 nonredundant metabolites at the MS2 level corresponding to 54 in positive and 41 in negative modes. The top-scoring metabolites from MS2query search with > 0.7 prediction score and rank 1 metabolites from MS2Compound were selected for docking with GDF15. The high-confidence metabolites in this study include Quercetin 3-O-[2''-*O*-b-d-glucopyranosyl]-a-l-rhamnopyranoside, 4-Hydroxyquinoline, Gallic acid 4-*O*-(6-galloylglucoside), Phosphatidylcholine among others. The metabolites identified from the search in MS2Compound are provided in Online Resource 2.

**Fig. 5:**
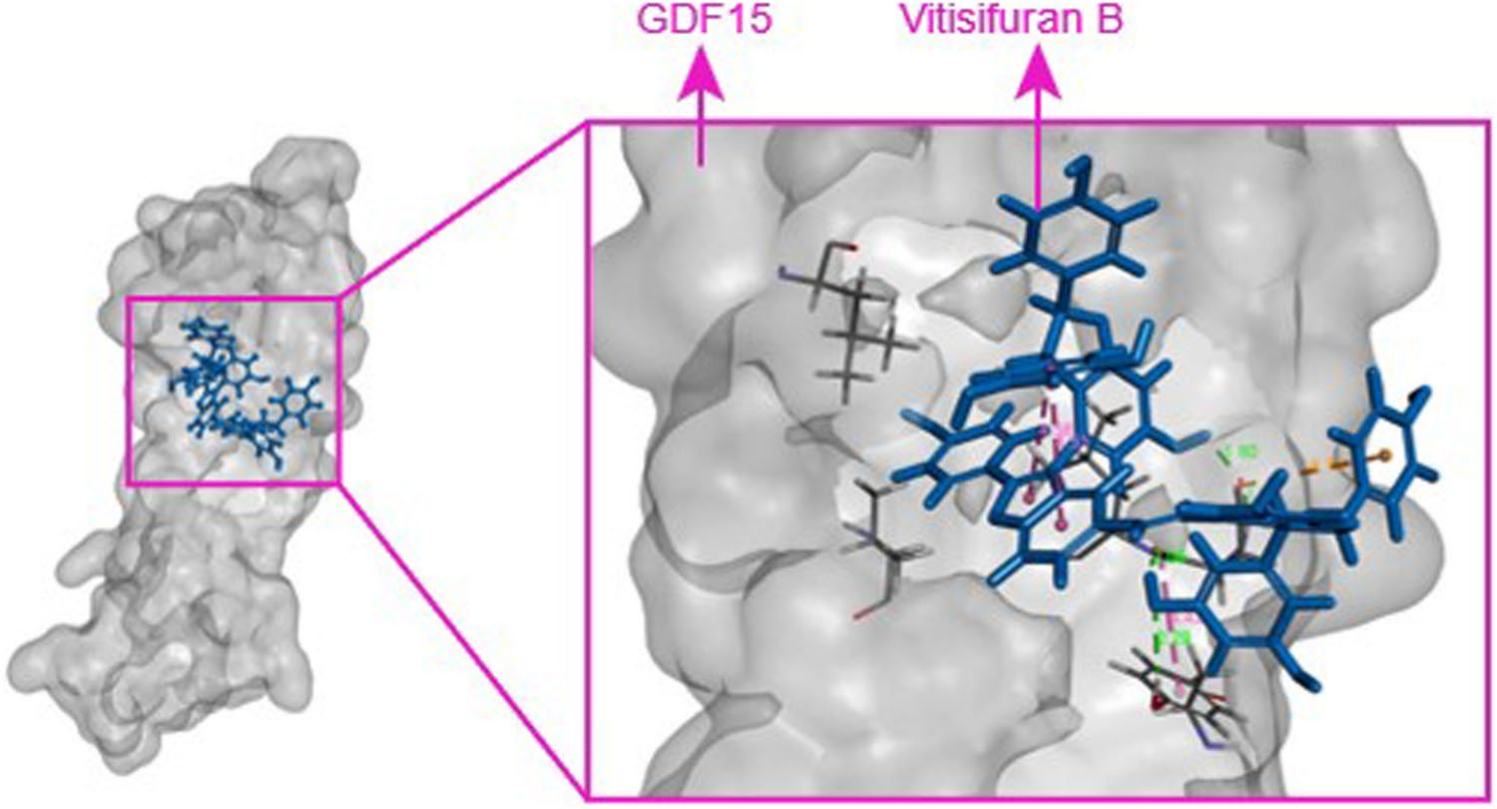
Visualization of 3D docked complex structure of Vitisifuran B with GDF15. The molecular docking model depicts the interaction between Vitisifuran B and GDF15. Vitisifuran B is shown to bind to the active site of GDF15, suggesting its potential as an inhibitor

Molecular docking was carried out to identify potential metabolites of ‘Kanchanara Guggulu’ that could bind to the active site of GDF15 to its receptor GFRAL, potentially contributing to its anticancer properties. The docking analysis revealed the successful docking of four compounds, with Vitisifuran B achieving the highest LibDock score (70.86) followed by Phosphatidylinositol lyso 18:1 (69.79), 5-(10-Nonadecenyl)resorcinol (65.57) and 3'-*N*-Acetyl-4'-*O*-(14-methylpentadecanoyl)fusarochromanone (33. 76). The ligand Vitisifuran B formed hydrogen bonds with Tyr279 and Asp299, along with additional interactions involving Asp299, Leu300, and Leu223. The docking results are summarized in Table 1, and the docked complex structure of Vitisifuran B with GDF15 is illustrated in Fig. 5.

**Table 1:** Molecular docking results of GDF15 with top 4 compounds

PubChem ID	IUPAC Name	Libdock score	Amino acids forming hydrogen bonds	Amino acids forming other interactions
131751783	Vitisifuran B	70.86	Tyr279, Asp299	Asp299, Leu300, Leu223
134756595	Phosphatidylinositol lyso 18:1	69.79	Cys240	Val235, Val237, Pro276, Ala302, Leu300, Val229, Pro232, Met282, Leu284, Trp225, Tyr279
131752736	5-(10-Nonadecenyl)-1,3-benzenediol	65.57	Gly224	Val229, Val235, Val237, Leu284, Leu223, Met282, Leu300, Leu220, Pro232, Trp225, Tyr297
101420967	3'-*N*-Acetyl-4'-*O*-(14-methylpentadecanoyl)fusarochromanone	33.76	Tyr279, Asp299	Asp299, Val235, Val237, Leu223, Leu300, Leu220, Val229, Pro232, Leu284, Trp225

The Highest LibDock score of Vitisifuran B suggests a favorable binding affinity, indicating that this metabolite may influence the GDF15-GFRAL interaction, potentially playing a role in its anticancer effects. Vitisifuran B is a member of the 2-arylbenzofuran flavonoid class of organic compounds, with unknown biological effects (https://doi.org/10.1016/S0040-4020(99)00039-3). Our study has demonstrated its potential in targeting GDF15, making this a key highlight of the research. Further experimental validations are required to confirm these findings. Docking studies were performed with four chemotherapeutic drugs against the same active site of GDF15. The results revealed that only doxorubicin successfully bound to the defined pocket, with a LibDock score of 68.29 and formed hydrogen bonds with Tyr279 and Asp299.

## Limitations of the study

Signaling pathway information is provided as a supplementary file. The curators and reviewers follow the established protocols for pathway curation, however, there is always room for confusion because of common alternate names for different proteins used by different laboratories. Each molecular reaction is sourced from different types of experiments from multiple laboratories. Model systems, experimental conditions, quality controls, and contexts of investigations may vary depending on the investigators and laboratories. The curators cannot have control over the quality of the way publicly available data is generated. We urge the biomedical community to participate in improving the pathway annotations by reporting any errors of omission/commission, which will help revise these signaling pathways.

## Conclusions

The depiction of molecular reactions induced by GDF15, which are reported from multiple laboratories under diverse experimental conditions into a single larger network of GDF15 as a signaling pathway resource, along with indications on its implications in different cancers, will provide a critical platform for designing further research investigations in this area. The accessibility of the GDF-15-mediated signaling pathway will assist researchers in biomedicine to comprehend the functions of various molecules controlled by GDF15 in the progression of cancers. We also demonstrated the pathway’s potential in functional enrichment studies and identified a compound of ‘Kanchanara Guggulu’ through molecular docking that could target the binding of GDF15 to GFRAL, filling a critical gap in available inhibitors. This work provides a foundation for further investigation into GDF15 signaling and its therapeutic targeting.

## Supplementary Information

Additional file1 (XLSX 428 KB)

Additional file2 (XLSX 1498 KB)

Additional file3 (DOC 37 KB)

Additional file4 (JPG 1418 KB)

Additional file5 (DOC 286 KB)

Additional file6 (DOCX 14 KB)

## Data Availability

The manually-assembled signaling pathway data is provided in the ‘.xlsx’ format (Online Resource 1). The pathway visualized using the PathVisio tool is provided as supplementary material in the file GDF15_pathway.doc. The corresponding '.gpml' format is available upon request and can be converted to international data exchange formats such as BioPAX, PSI-MI, and SBML. The analyzed metabolomics data is provided in ‘.xlsx’ format (Online Resource 2).

## Abbreviations

ACSL1: Acyl-CoA synthetase long chain family member 1
AKT1: AKT serine/threonine Kinase 1
BC: Breast cancer
CDH1: Cadherin 1
CDH2: Cadherin 2
CDKN1A: Cyclin dependent kinase inhibitor 1A
CPT1A: Carnitine palmitoyltransferase 1A
CPT1B: Carnitine palmitoyltransferase 1B
CPT2: Carnitine palmitoyltransferase 2
CRC: Colorectal cancer
EGFR: Epidermal growth factor receptor
EMT: Epithelial-mesenchymal transition
ERBB2: Erb-B2 receptor tyrosine kinase 2
GDF15: Growth differentiation factor 15
GFRAL: GDNF family receptor alpha like
HNC: Head and neck cancer
IGF1R: Insulin like growth factor 1 receptor
MAPK1: Mitogen-activated protein kinase 1
MAPK14: Mitogen-activated protein kinase 14
MAPK3: Mitogen-activated protein kinase 3
NDRG1: N-Myc Downstream Regulated 1
PPARGC1A: PPARG coactivator 1 alpha
PTM: Post-translational modification
rhGDF15: Recombinant human GDF15
Ser: Serine
SERPINB5: Serpin family B member 5
SMAD2: SMAD family member 2
SMAD3: SMAD family member 3
SNAI1: Snail family transcriptional repressor 1
SOX2: SRY-Box transcription factor 2
TGFBR2: Transforming growth
