# Supplementary material for: Mapping growth differentiation factor-15 (GDF15)-mediated signaling pathways in cancer: insights into its role across different cancer types
Source: Discov Oncol. 2025 Mar 25;16:386. doi: 10.1007/s12672-025-02121-1 (PMC11933546; doi:10.1007/s12672-025-02121-1)
Supplement: Supplementary file 3 — Additional file3 (DOC 37 KB) [file 12672_2025_2121_MOESM3_ESM.doc]

**Online Resource 3: Table summarizing the software, tools, and databases used in the study**

| **Sl. No.** | **Software/ tools/ Databases used** | **Purpose** | **Output** |
| --- | --- | --- | --- |
| 1. | PubMed | Literature search for experimental studies on GDF15-mediated signaling | Curated list of signaling events linked to GDF15 |
| 2. | PathBuilder, NetPath, and NetSlim | Data curation and pathway mapping reference | Formatted Excel sheets with curated signaling events |
| 3. | PathVisio | Manual drawing and visualization of the GDF15 pathway map | GDF15 pathway map in gpml format |
| 4. | STRING | Analysis of protein-protein interactions | Interaction network for colorectal cancer (CRC) and breast cancer (BC) -associated proteins |
| 5. | KEGG and WikiPathways | Reference for CRC and BC pathway data. Identify proteins not annotated in databases. | List of proteins, including those with incomplete annotations, in the CRC and BC pathways (Figure 3a and Figure S1a) |
| 6. | SRplot | Analysis of TCGA colon adenocarcinoma mutation data using MAF Oncoplot | Mutation profiles of CRC and BC-associated proteins (Figure 3b and Figure S1b) |
| 7. | MSConvert | Conversion of .wiff files to mzML format for further analysis in MZmine | Converted mzML files from raw .wiff files for further data analysis in MZmine |
| 8. | MZmine (Version 2.53) | Preprocessing of metabolomics data to extract features and align retention times | CSV files with peak areas, retention times, m/z values, and feature IDs |
| 9. | PlantCyc and HMDB | Metabolite annotation | Annotated metabolite list from MS2 data |
| 10. | MS2query | MS2 spectral comparison to identify best-matching metabolites | Top-scoring metabolites taken for molecular docking against GDF15 |
| 11. | Discovery Studio 2022 | Preparation of GDF15 structure and metabolites for docking | Docking-ready structures of GDF15 and metabolites |
| 12. | PubChem | Retrieval of metabolite structures | Ligand structures for docking |
