# Supplementary figures and images for: Mapping growth differentiation factor-15 (GDF15)-mediated signaling pathways in cancer: insights into its role across different cancer types
Source: Discov Oncol. 2025 Mar 25;16:386. doi: 10.1007/s12672-025-02121-1 (PMC11933546; doi:10.1007/s12672-025-02121-1)

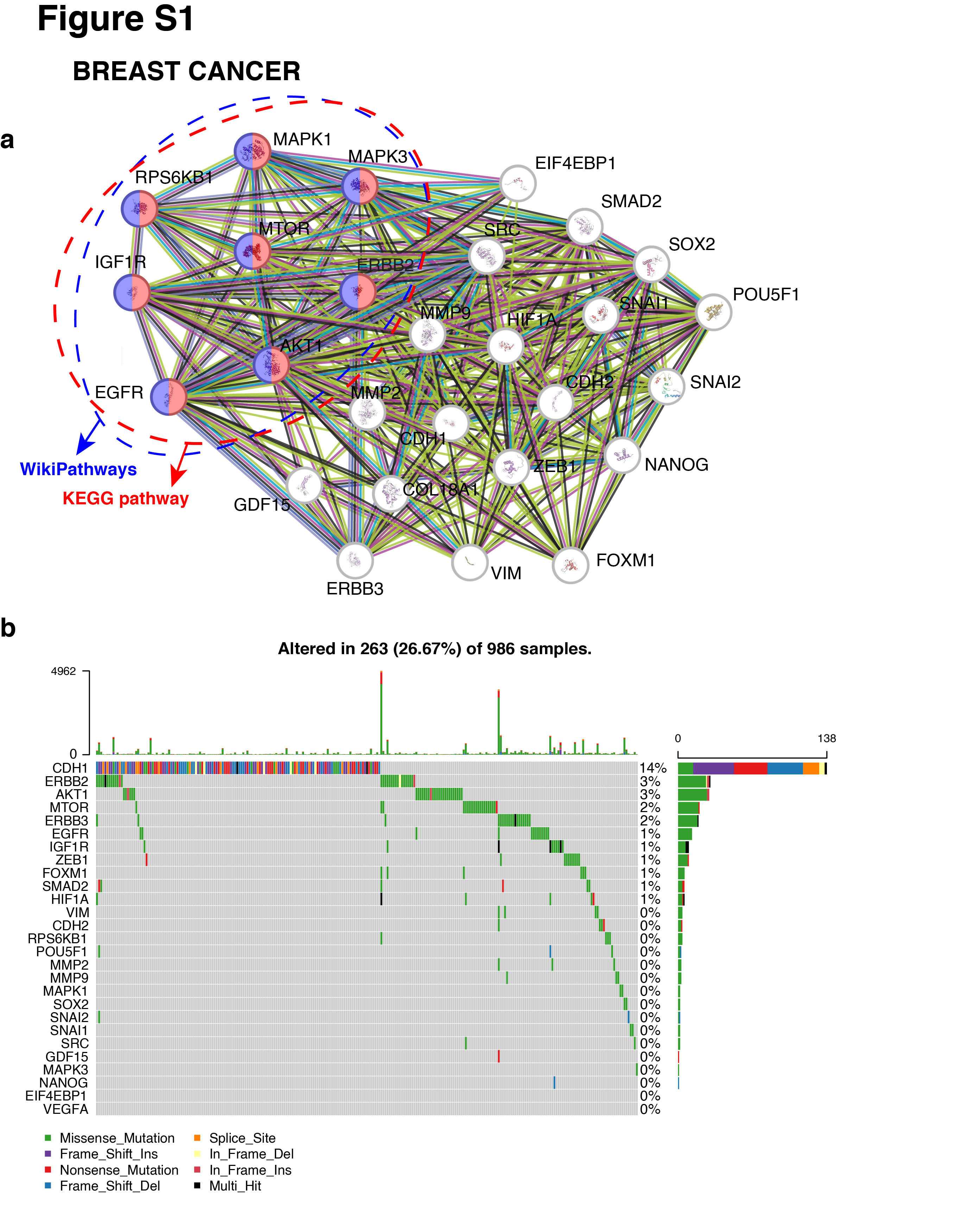

Supplement: Supplementary file 4 — Additional file4 (JPG 1418 KB) [file 12672_2025_2121_MOESM4_ESM.jpg]

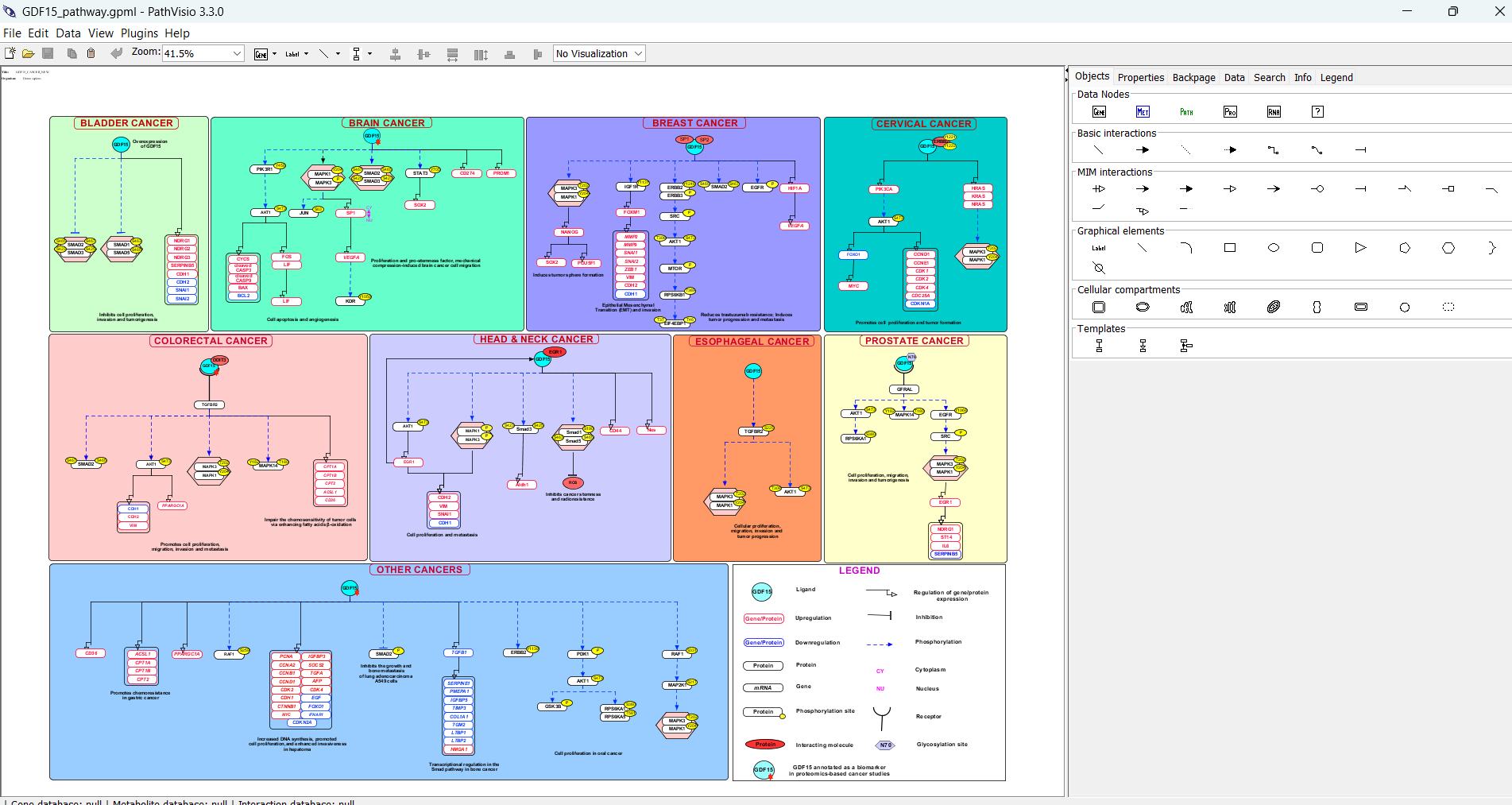

Supplement: Supplementary file 5 — Additional file5 (DOC 286 KB) [file 12672_2025_2121_MOESM5_ESM.doc]
