# Supplementary material for: Mapping growth differentiation factor-15 (GDF15)-mediated signaling pathways in cancer: insights into its role across different cancer types
Source: Discov Oncol. 2025 Mar 25;16:386. doi: 10.1007/s12672-025-02121-1 (PMC11933546; doi:10.1007/s12672-025-02121-1)
Supplement: Supplementary file 6 — Additional file6 (DOCX 14 KB) [file 12672_2025_2121_MOESM6_ESM.docx]

**Supplementary Material**

**Figure Legend**

**Fig. S1 Comparison of GDF15 pathway proteins associated to breast cancer with existing pathway databases and TCGA mutational data**

(a) The protein interaction network of proteins from the GDF15 pathway map with those found in the breast cancer (BC) pathway. Proteins marked with circles represent hits in KEGG (red) and WikiPathways (blue). (b) The MAF Oncoplot illustrates mutation frequencies of BC proteins in TCGA data for breast invasive carcinoma.
